# A bacterial dual positive and negative selection system for dCas9 activity

**DOI:** 10.1371/journal.pone.0269270

**Published:** 2022-06-03

**Authors:** Shaun Spisak, Brett O’Brien, Marc Ostermeier

**Affiliations:** 1 Chemistry-Biology Interface Graduate Program, Johns Hopkins University, Baltimore, MD, United States of America; 2 Department of Chemical & Biomolecular Engineering, Johns Hopkins University, Baltimore, MD, United States of America; Hirosaki University Graduate School of Medicine, JAPAN

## Abstract

The engineering of switchable or activatable dCas9 proteins would benefit from a single system for both positive and negative selection of dCas9 activity. Most systems that are used to interrogate dCas9 libraries use a fluorescent protein screen or an antibiotic selection for active dCas9 variants. To avoid some of the limitations of these systems, we have developed a single system capable of selecting for either active or inactive dCas9 variants. *E*. *coli* expressing active dCas9 variants are isolated in the positive selection system through growth in the presence of ampicillin. The negative selection can isolate cells lacking dCas9 activity through two separate mechanisms: growth in M9 minimal media or growth in media containing streptomycin. This system is capable of enriching for rare dCas9 variants up to 9,000-fold and possesses potential utility in directed evolution experiments to create switchable dCas9 proteins.

## Introduction

Protein libraries allow for the creation of novel protein variants with increased catalytic activity, thermostability, alternative binding specificities, and entirely new functions [1]. Library creation methods such as random mutagenesis, recombination, and domain insertion can generate thousands to millions of these protein variants. However, only a small subset of these variants possesses the desired function. Therefore, systems for performing high-throughput screens or selections are necessary to effectively sample the broad area of sequence space that is covered by protein libraries [1].

An attractive target for protein engineering is the CRISPR associated protein, Cas9, which binds and cleaves DNA in a sequence-specific manner through base-pairing interactions with a short guide-RNA molecule (gRNA) that Cas9 complexes with [2]. We are particularly interested in the nuclease-null variant of this protein, referred to as dCas9, which can repress gene expression by blocking transcription of RNA polymerase [3]. Cas9 and dCas9 have been engineered to recognize alternative binding targets [4], modulate activity in the presence of light [5] or a small molecule [6], activate transcription of a gene of interest [7], and possess other desired properties [8]. We intend to develop dCas9-based protein switches that will modulate their repressive activity on gene expression in response to a small molecule input. As with other existing dCas9 switches, the increased temporal control of dCas9 activity will allow for the creation of more complex gene circuits and for more flexible control of expression of genes targeted by dCas9. There are unique challenges associated with developing a protein switch compared to other CRISPR/Cas9 engineering projects. Most significantly, the high-throughput screening system must be able to identify both active and inactive protein variants under different conditions (e.g. with and without activating-ligand).

Green fluorescent protein (GFP) [9] has been one of the most used reporters of dCas9 activity. In these systems, a *GFP*-targeting gRNA causes low cell fluorescence if a functional dCas9 is present. This reporter system is attractive to use, as measuring whole-cell fluorescence is fast, simple, and noninvasive and can also be used for screening large protein libraries when used in combination with Fluorescence Activated Cell Sorting (FACS). Importantly, this system can be used to identify both low and high activity dCas9 variants. However, despite its frequent use, this system has limitations. Collecting cells with low levels of fluorescence can be difficult due to low resolution, and selection for loss-of-function (loss of fluorescence) is inherently prone to false positives such as loss of the GFP gene or inactivating mutations. Additionally, performing FACS requires specialized and expensive instrumentation that is not always readily available to some researchers. Reporter systems other than GFP have infrequently been used to screen dCas9 libraries. However, these systems are situational (e.g. Phage-Assisted Continuous Evolution [4]) or specific to dCas9 fused to activators of gene expression, which could only be used for positive selection for dCas9 activity [7]. We sought to develop a single selection-based system that can be used to isolate either low or high activity dCas9 variants and lacks some of the limitations of GFP-based systems. The system we developed uses ampicillin resistance as a positive selection and streptomycin resistance as a negative selection for dCas9 activity. Our dual positive and negative selection is capable of enriching for cells lacking or containing active dCas9 by up to 9,000-fold and will, in principle, be useful for isolating conditionally active dCas9 variants in large protein libraries.

## Materials and methods

### Media and reagents

All growth experiments were performed in LB media (10 g/L tryptone, 5 g/L yeast extract, and 10 g/L sodium chloride) at 37°C unless otherwise specified. Agar was added to 1.5% for solid media experiments. M9 minimal media contained 6 g/L dibasic sodium phosphate, 3 g/L monobasic potassium phosphate, 0.5 g/L sodium chloride, 1 g/L ammonium chloride and was supplemented with 1 mM magnesium sulfate, 0.1 mM calcium chloride, and 1% glycerol. Noble agar was added to 2% for solid media experiments in M9 minimal media. All enzymes were obtained from New England Biolabs.

### Plasmids and strains

A list of strains and plasmids is provided in S1 Table in S1 File. All experiments were performed in *E*. *coli* strain K12 MG1655 [10] and all cloning was performed in *E*. *coli* strain NEB5α unless otherwise specified. All plasmids were constructed using traditional cloning techniques.

### Minimum inhibitory concentration assays

MG1655 cells were grown in LB supplemented with the appropriate antibiotic for plasmid maintenance; 35 μg/mL chloramphenicol for pdCas9 and 50 μg/mL spectinomycin for the pSelect plasmids. While the cells were growing, LB-agar plates with ampicillin or streptomycin in increasing two-fold amounts were prepared. The plates were supplemented with antibiotic(s) for plasmid maintenance and 2 nM anhydrotetracycline (aTc), the dCas9 expression inducer, as necessary. The ampicillin and streptomycin were prepared fresh for each experiment. After growing for approximately eight hours, the cells were diluted 10,000-fold in LB, spread on each plate, and incubated overnight. The minimum inhibitory concentration of an antibiotic was determined by counting the number of colonies on each plate. The concentration of antibiotic at which the number of colonies on the plate was less than 5% of the total number of colonies on antibiotic-free control plates was determined to be the minimum inhibitory concentration for that antibiotic. Each experiment was performed in duplicate.

### Fluorescence assay

Fluorescent MG1655 cells [3] harboring pSelect-6 with and without pdCas9 were grown overnight in LB supplemented with the appropriate antibiotics for plasmid maintenance. In the next morning, the cells were diluted to an OD600 of 0.001 in LB supplemented with 2 nM aTc and antibiotics as necessary and incubated for six hours. For each culture, three aliquots were assayed in a 96-well plate. A Spectramax M3 plate reader was used to measure the OD600 and fluorescence of each aliquot. The excitation and emission wavelengths were 485 and 525 nm respectively. The average of the relative fluorescence (GFP/OD600) from each well was used as a measure of GFP expression. This experiment was repeated twice on separate days.

### Phenylalanine auxotrophy assay

MG1655 cells harboring pdCas9 and pSelect-2 or pSelect-7 were grown overnight in LB supplemented with chloramphenicol and spectinomycin. In the next morning, portions of the cells were pelleted, washed once with M9, and resuspend in M9. The cells were diluted to an OD600 of 0.1 in M9 and then further diluted in a series of six ten-fold dilutions. Three μL of each dilution were spotted on LB-agar and M9-agar plates. The M9-agar plates were supplemented with 2 nM aTc and 10 μg/mL phenylalanine as necessary. The plates were incubated at 37°C and imaged at 24 hours (LB) or 72 hours (M9).

### Mock enrichment assays

MG1655 cells harboring pSelect-9 and either pdCas9 or pEV were grown for approximately eight hours in LB supplemented with chloramphenicol and spectinomycin. In experiments to test enrichment for cells exhibiting dCas9 activity, cells harboring pdCas9 were mixed with cells harboring pEV in 1:1000 and 1:10,000 ratios by volume. Each mixture of cells was further diluted 10,000-fold in LB and spread on LB-agar plates supplemented with chloramphenicol, 2 nM aTc, and 256 μg/mL ampicillin. In experiments to test enrichment for cells lacking dCas9 activity, cells harboring pEV were similarly diluted in cells harboring pdCas9 and spread on LB-agar plates supplemented with chloramphenicol, 2 nM aTc, and 256 μg/mL streptomycin. For each mixture, ten colonies were picked and grown overnight in LB and their plasmid DNA was isolated using a QIAprep Miniprep kit. Plasmid DNA (750 ng) was incubated with BglII at 37°C for three hours. A total of 500 ng of pdCas9, pEV, and pSelect-9 DNA were digested as controls. The DNA was separated on a 0.8% agarose gel at 90 V for one hour. Each gel was imaged using ethidium bromide and UV light and cells with pEV or pdCas9 were identified by the digestion pattern. These experiments were repeated following the same procedure in NEB5α cells.

To enhance plasmid enrichment, colonies from the 10,000-fold enrichment experiments were collected in 1 mL of LB per plate. Approximately 500 colonies were collected in total from each experiment. A portion of the cells was immediately spread on plates containing the same antibiotics as before. Additionally, plasmid DNA from colonies from the dCas9 enrichment experiment was isolated and treated with ApaLI and T5 exonuclease at 37°C for two hours, which will degrade the pSelect-9 plasmid but not the pdCas9 plasmid. The DNA was collected using a Zymo DNA Clean and Concentrator Kit and transformed in MG1655 cells along with fresh pSelect-9 plasmid. Approximately 2000 colonies were collected from the resulting plates and spread on fresh agar plates containing chloramphenicol, 2 nM aTc, and 256 μg/mL ampicillin. Ten colonies from each experiment were screened following the same procedure as above.

## Results and discussion

### Single guide RNA positive selection

**Fig 1:**
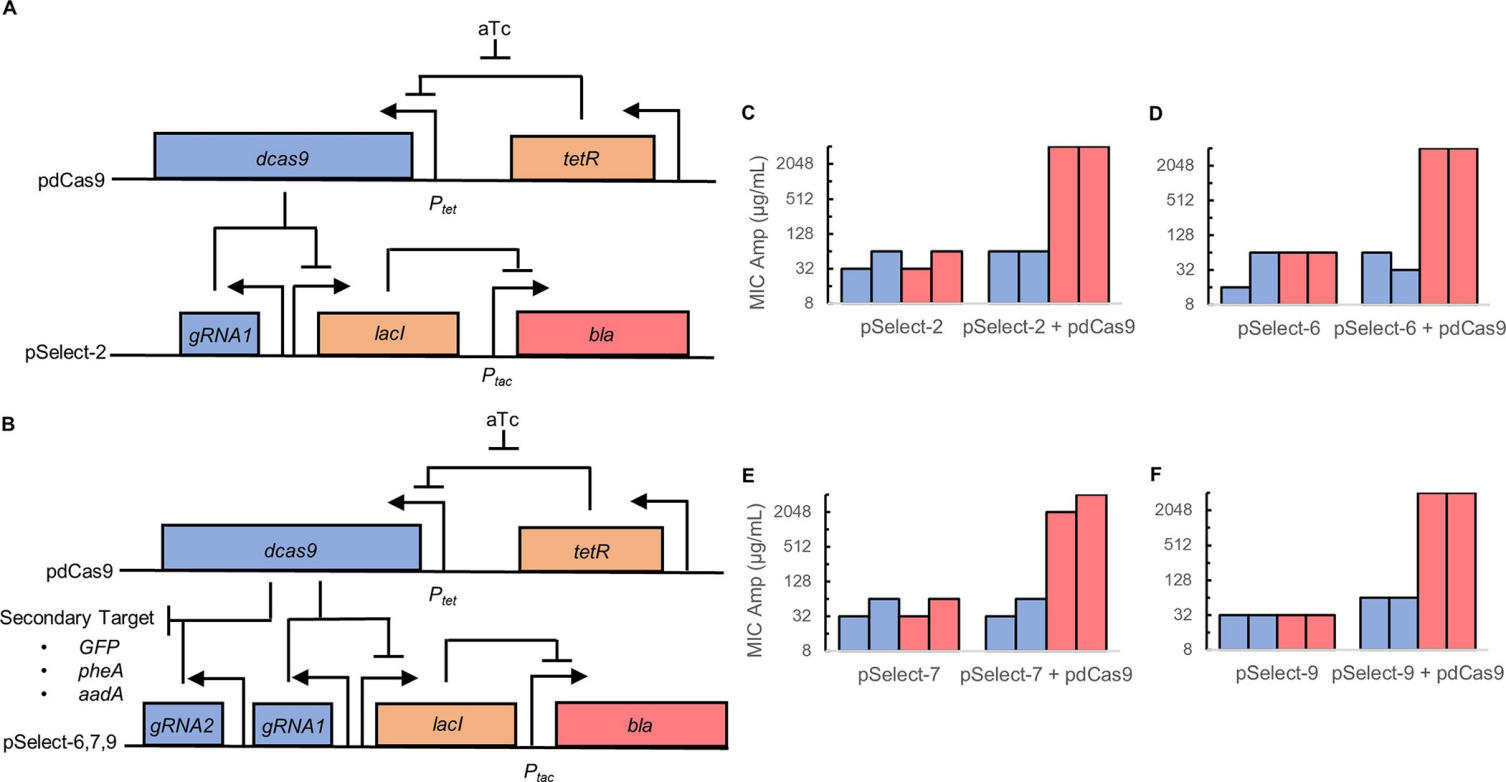
Positive selection system for dCas9 activity. (A) Schematic representation of the single gRNA positive selection circuit. LacI represses expression of β-lactamase (*bla*) and is repressed by dCas9 complexed with a LacI-targeting gRNA. Full plasmid maps provided as S1 Fig in S1 File. (B) Schematic representation of the dual gRNA selection circuit. An additional gRNA is incorporated into the selection plasmid, allowing dCas9 to target a second gene. (C-F) Replica minimum inhibitory concentration assays. Each experiment was performed in the absence (left bars) and presence (right bars) of the pdCas9 plasmid and in the absence (blue bars) and presence (red bars) of 2 nM aTc. Each assay was performed twice.

We developed a two-plasmid system for selecting for functional dCas9. The first plasmid, pdCas9, contained the *dCas9* gene under the control of the tetracycline promoter (Fig 1A). The second plasmid, pSelect-1, contained the β-lactamase gene (*bla)*, our reporter gene for dCas9 function that confers ampicillin resistance. This plasmid also contained the *E*. *coli lacI* gene/promoter and a guide RNA specific for the *lacI* gene constitutively expressed under the *J23119* promoter. LacI repressed the *tac* promoter that controlled expression of β-lactamase. To increase *lacI* expression, we replaced the GTG alternative start codon that occurs naturally on *lacI* with the standard ATG start codon to make pSelect-2. In the absence of dCas9, LacI repressed expression of the *β-lactamase* gene, leading to a low minimum inhibitory concentration (MIC) of ampicillin of 32–64 μg/mL on LB-agar (Fig 1C). When dCas9 expression is induced with anhydrotetracycline (aTc), expression of LacI is repressed by dCas9, alleviating repression of β-lactamase. dCas9 expression increased the MIC of ampicillin approximately 32-fold to 2048 μg/mL.

### Dual guide RNA positive selection

The above system was designed to isolate cells expressing active dCas9 variants. However, in certain cases, such as in the creation of a dCas9 switch, there is a need to isolate cells that express inactive dCas9 variants. The single guide RNA system of pSelect-2 cannot be used for these negative selections. Therefore, we identified three secondary gene targets: green fluorescent protein (*GFP*) [9], prephenate dehydratase (*pheA*) [11], and aminoglycoside adenlylytransferase (*aadA*) [12], to be used in a negative selection. We added a second gRNA targeting each the above three genes to pSelect-2 to create pSelect-6, pSelect-7, and pSelect-9, respectively (Fig 1B, S1 Fig in S1 File). We first verified that the expression of these second gRNA did not diminish repression of the *lacI* gene by reducing the number of dCas9 molecules available to repress LacI expression. Cells harboring these dual gRNA plasmids showed a similarly high resistance to ampicillin in the presence of dCas9 (Fig 1C–1F).

### Dual guide RNA negative selection

**Fig 2:**
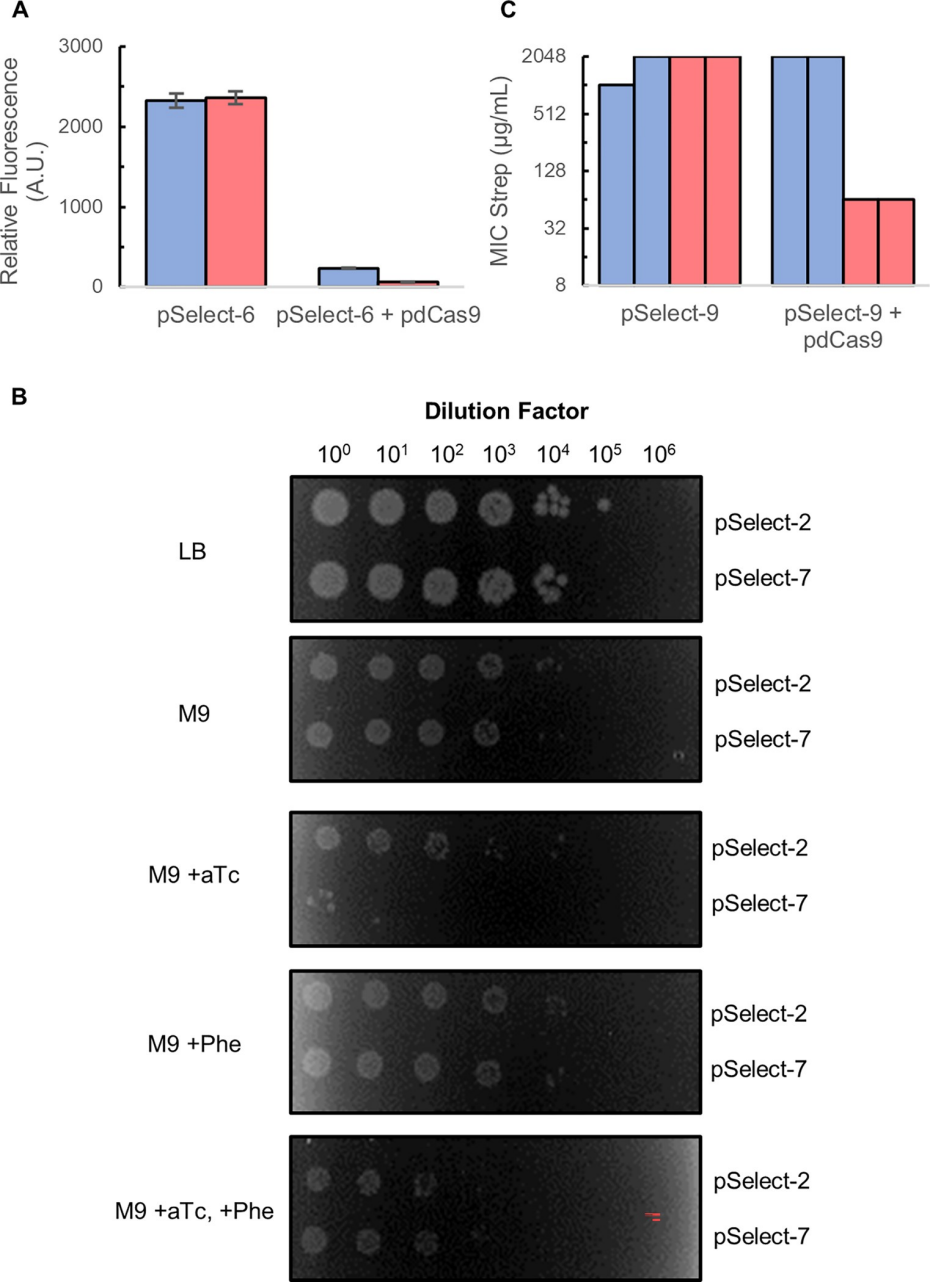
Negative selection system for dCas9 activity. (A) Relative GFP fluorescence after six hours of growth of MG1655 *nfsA*::*(mRFP*, *sfGFP*, *Kan*^*R*^*)* cells [3]. Each experiment was performed in the absence (left bars) and presence (right bars) of the pdCas9 plasmid and in the absence (blue bars) and presence (red bars) 2 nM aTc. Error bars represent the standard deviation (n = 3). (B) MG1655 cells spotted on LB-agar and M9-agar. The media along with any supplements it contained is indicated along the column to the left of the plates. aTc was added to a concentration of 2 nM, and phenylalanine was added to a concentration of 10 μg/mL. The selection plasmid present is indicated along the column to the right side of the plates and the dilution factor of each spot is indicated above the plates. (C) Replica minimum inhibitory concentration assays. Each assay was performed twice. Each experiment was performed in the absence (left bars) and presence (right bars) of the pdCas9 plasmid and in the absence (blue bars) and presence (red bars) 2 nM aTc.

We next tested the ability of the second gRNAs to function as reporters for the absence of dCas9 activity. The first plasmid, pSelect-6, expresses a gRNA that targets chromosomally expressed GFP. As expected, GFP expression was approximately 40-fold higher in the absence of dCas9 than in its presence (Fig 2A). Even the leaky expression of dCas9 in the absence of aTc was enough to cause significant repression of GFP expression. Based on the data we obtained with this plasmid, we anticipated that our dual gRNA approach should effectively provide positive and negative selection systems.

To move away from fluorescence-based screens of dCas9 activity, we next examined selection plasmids that used alternative secondary targets. The first of these plasmids, pSelect-7, expresses a gRNA that targets the *pheA* gene on the *E*. *coli* chromosome. This gene encodes a protein that is responsible for catalyzing the first committed step in phenylalanine biosynthesis. Cells that lack this protein can grow in nutrient-rich growth media, such as LB. However, when incubated in nutrient-poor media, such as M9 minimal media, cells that lack PheA are unable to grow unless phenylalanine is supplemented to the media. Therefore, in principle, cells that lack active dCas9 should be able to grow on M9 while cells that have active dCas9 and the *pheA*-repressing gRNA should not. To test this hypothesis, cells harboring the pdCas9 plasmid and either pSelect-2 or -7 were spotted on LB-agar and M9-agar plates in a series of ten-fold dilutions. Both strains grew equally well on LB and on M9 media in the absence of induction of dCas9 expression (Fig 2B). However, when dCas9 was induced by adding aTc to the M9 media, cells containing the *pheA*-repressing gRNA grew markedly worse than cells lacking this gRNA. Supplementing the media with Phe alleviated this growth differential. We conclude that cells containing pSelect-7 grow better on minimal media in the absence of a functional dCas9.

The final dual gRNA selection plasmid, pSelect-9, targets aminoglycoside adenlylytransferase (*aadA*). This gene is expressed constitutively on the plasmid, confers resistance to streptomycin, and is used for plasmid maintenance outside of selection experiments. In principle, active dCas9 would repress expression of the gene, leading to low streptomycin resistance, and creating conditions in which only cells that lack active dCas9 are able to grow in the presence of this antibiotic. Indeed, MIC assays showed that cells harboring both the pdCas9 and pSelect-9 plasmids had a MIC of streptomycin of 64 μg/mL in the presence of aTc (Fig 2C). This MIC is approximately 32-fold lower than the MIC of streptomycin in cells that lack active dCas9, which is 1024–2048 μg/mL. The data support the idea that cells lacking dCas9 activity could be selected from cells containing dCas9 activity by plating at a sufficiently high concentration of Strep.

### Mock enrichment assay

The primary purpose of these dual gRNA selection systems was to aid in the engineering of conditionally-active dCas9 variants. We devised a set of mock-library experiments to test the ability of the Strep-based system to enrich for cells expressing active or inactive dCas9. Each experiment used *E*. *coli* harboring the pSelect-9 plasmid and either the pdCas9 plasmid or its backbone without the *dCas9* gene, referred to as pEV. Liquid cultures of each of these strains were grown from frozen glycerol stocks for approximately eight hours until the media was saturated with cells. To test the ability of the system to enrich for active dCas9, we made 1000:1 and 10,000:1 mixtures of pEV:pdCas9-harboring cells and spread them on agar plates containing 256 μg/mL ampicillin. Ten colonies from each of these plates were picked and grown overnight. Their plasmid DNA was isolated, digested with BglII, and separated on an agarose gel. Enrichment of the 1000-fold diluted cells was highly successful (Table 1). All ten colonies screened from the dCas9 enrichment contained the pdCas9 plasmid. However, experiments using the 10,000-fold diluted cells revealed the enrichment limit. Only 20% (2/10) of the colonies screened contained the desired plasmids. In addition, unexpected digestion patterns for the plasmid DNA from some colonies suggested the presence of unwanted recombination or mutations in these false positives (S2C Fig in S1 File).

**Table 1:** Mock enrichment of active and inactive dCas9.

			Post-selection cells containing plasmid			
Ratio prior to selection (pEV:pdCas9)	Strain	Selection conditions	pEV	pdCas9	Other^a^	Enrichment of desired plasmid
1,000:1	MG1655	256 μg/ml Amp (once)	0% (0/10)	100% (10/10)	0% (0/10)	>900-fold
10,000:1	MG1655	256 μg/ml Amp (once)	50% (5/10)	20% (2/10)	30% (3/10)	2,000-fold
10,000:1	MG1655	256 μg/ml Amp (twice)	30% (3/10)	60% (6/10)	10% (1/10)	6,000-fold
10,000:1	MG1655	256 μg/ml Amp (twice + ApaL1 digestion)	0% (0/10)	100% (10/10)	0% (0/10)	>9,000-fold
10,000:1	NEB5α	256 μg/ml Amp (once)	90% (9/10)	0% (0/10)	10% (1/10)	none observed
1:1,000	MG1655	256 μg/ml Strep (once)	90% (9/10)	10% (1/10)	0% (0/10)	900-fold
1:10,000	MG1655	256 μg/ml Strep (once)	20% (2/10)	50% (5/10)	30% (3/10)	2,000-fold
1:10,000	MG1655	256 μg/ml Strep (twice)	70% (7/10)	0% (0/10)	30% (3/10)	7,000-fold
1:10,000	NEB5α	256 μg/ml Strep (once)	90% (9/10)	10% (1/10)	0% (0/10)	9,000-fold

^a^Plasmids having a digestion pattern that does not match pEV or pdCas9

Collecting the colonies from the 10,000-fold diluted plates and spreading them a second time on Amp partially increased the frequency of pdCas9 containing cells from 20% to 50%. This suggests that for some of the false positives, growth on ampicillin on the first plating was likely due to stochastic variation rather than a mutation conferring growth on ampicillin in the absence of functional dCas9. For the remaining false positives, we hypothesized that most mutations that could confer ampicillin resistance to cells harboring the pEV plasmid would occur on the pSelect-9 plasmid. For example, mutations that disrupt LacI expression or *lac* operon binding could lead to increased expression of β-lactamase, removing the need for active dCas9. To test this hypothesis, we collected all colonies *en masse* from the 10,000:1 ratio plates and isolated their plasmid DNA. The DNA was then treated with ApaLI (which does not cut pdCas9, but does cut pSelect-9 at four sites) and T5 exonuclease to selectively degrade the pSelect-9 plasmid. The digested DNA was then transformed into *E*. *coli* along with fresh pSelect-9. The transformants were collected and spread on agar plates containing ampicillin. A screen of the colonies from these plates showed that 100% (10/10) of them harbored pdCas9 (Table 1). We also hypothesized that enrichment could be affected by cell type. We repeated the 10,000-fold dilution selection experiment using NEB5α cells, which lack functional *endA* and *recA* genes and are less prone to recombination. However, the use of NEB5α cells did not improve the enrichment of the pdCas9 plasmid over that obtained using the *endA*^+^ *recA*^+^ MG1655 cells.

Similarly, to test the enrichment of cells lacking dCas9, 1:1000 and 1:10,000 mixtures of pEV:pdCas9-harboring cells were spread on agar plates containing 256 μg/mL streptomycin. Analogous to the positive selection, the 1:1000 enrichment test worked very well, but most of the plasmids from the 1:10,000 enrichment test were not the desired plasmid (Table 1). Like the positive selection, a second plating under selective conditions (in this case plating with Strep) improved the frequency of desired plasmids from 20% (2/10) to 70% (7/10). In contrast to the positive selection, the use of NEB5α instead of MG1655 cells improved the frequency of desired plasmids from 20% to 90%, alleviating the need to retransform plasmids with fresh pSelect-9 plasmid. These experiments show that this dual gRNA system can be used to isolate rare dCas9 variants (< 0.1% of the total population) in as few as one round of spreading cells on agar.

Our antibiotic positive selection system is comparable to the one employed by Ho et al. to produce a dCas9-based transcriptional activator [7]. Using a single antibiotic, their false positive breakthrough rate was 1 in 10^5^, which might suggest that their system would be about 10-fold better than ours at enriching functional dCas9 variant, as we observed about a 10^4^-fold enrichment. However, they do not report an enrichment rate and our experiments use the more “library-like” conditions by including true positives. The presence of true positives in our experiments could allow false positives to occur more readily. They were able to further decrease their false positive breakthrough rate to 1 in 10^6^ by using two antibiotic positive selections simultaneously. Should the need arise, our system could be adapted to incorporate a second simultaneous antibiotic selection to increase efficacy.

Our system can potentially be used to identify dCas9 variants that display switching behavior. In principle, this would be done in as few as three steps (S3 Fig in S1 File). First, a library of variants, for example a series of random domain insertions, is generated and co-transformed with either pSelect-7 or -9. Next, the library is put through a single positive selection on ampicillin to identify active variants. The surviving cells would then be collected and put through a single negative selection on streptomycin in the presence of the desired input signal (i.e. light, small molecule, etc.). In principle, the remaining cells would express dCas9 switches, which can then be further validated and characterized. Because the pSelect-7 and -9 plasmids are designed to contain the components for both positive and negative selections, no more than a single transformation step should be necessary. However, for libraries in which active variants are exceedingly rare, additional rounds of positive and negative selections can be performed with fresh pSelect plasmid, as we have demonstrated. This would increase the frequency of desired variants and decrease the frequency of undesired mutants associated with other components of the plasmid, such as the antibiotic resistance genes.

Though many engineered dCas9 variants are designed to be used in human cell lines, this system is optimized for using bacterial codon optimized dCas9. As there is evidence that mammalian codon optimized dCas9 is expressed in lower amounts in certain *E*. *coli* strains [13], this represents a potential limitation of our system. We therefore recommend that variants initially be designed and tested using bacterial codon optimized dCas9, followed by optimizing the variant for mammalian expression as necessary. A further limitation is that our system does not evaluate dCas9 specificity, and thus selected variants might be more promiscuous in their off-target effects. If so, other bacterial systems designed to evaluate and evolve specificity might be used [14].

## Conclusions

This system can be used to perform both positive and negative selections for dCas9 activity without additional cloning or transformation steps between selections. These selection approaches are also unique in that they do not rely on FACS to isolate variants and therefore do not have limitations associated with this technique. However, these selections are still comparable in efficacy to other previously used selection systems. For these reasons, this system may be useful for the directed evolution of dCas9 variants, in particular ones that act as protein switches.

## Supporting information

S1 File Contains S1-S3 Figs and S1 Table along with captions. (PDF)

S1 Data Contains raw optical density and fluorescence data used to create Fig 2A. (XLSX)

S1 Raw images Uncropped gels used in S2 Fig in S1 File with lanes numbered and labeled. (PDF)

## References

[1] Packer MS, Liu DR. Methods for the directed evolution of proteins. Nat Rev Genet. 2015;16: 379–394. doi: 10.1038/nrg3927 26055155

[2] Jinek M, Chylinski K, Fonfara I, Hauer M, Doudna JA, Charpentier E. A Programmable Dual-RNA–Guided DNA Endonuclease in Adaptive Bacterial Immunity. Science. 2012;337: 816–822. doi: 10.1126/science.1225829 22745249 PMC6286148

[3] Qi LS, Larson MH, Gilbert LA, Doudna JA, Weissman JS, Arkin AP, et al. Repurposing CRISPR as an RNA-Guided Platform for Sequence- Specific Control of Gene Expression. Cell. 2013;152: 1173–1183. doi: 10.1016/j.cell.2013.02.022 23452860 PMC3664290

[4] Hu JH, Miller SM, Geurts MH, Tang W, Chen L, Sun N, et al. Evolved Cas9 variants with broad PAM compatibility and high DNA specificity. Nature. 2018;556: 57–63. doi: 10.1038/nature26155 29512652 PMC5951633

[5] Nihongaki Y, Kawano F, Nakajima T, Sato M. Photoactivatable CRISPR-Cas9 for optogenetic genome editing. Nat Biotechnol. 2015;33: 755–760. doi: 10.1038/nbt.3245 26076431

[6] Oakes BL, Nadler DC, Flamholz A, Fellmann C, Staahl BT, Doudna JA, et al. Profiling of engineering hotspots identifies an allosteric CRISPR-Cas9 switch. Nat Biotechnol. 2016;34: 646–651. doi: 10.1038/nbt.3528 27136077 PMC4900928

[7] Ho H, Fang JR, Cheung J, Wang HH. Programmable CRISPR‐Cas transcriptional activation in bacteria. Mol Syst Biol. 2020;16: 1–12. doi: 10.15252/msb.20199427 32657546 PMC7356669

[8] Adli M. The CRISPR tool kit for genome editing and beyond. Nat Commun. 2018;9. doi: 10.1038/s41467-018-04252-2 29339724 PMC5770429

[9] Zimmer M. Green fluorescent protein (GFP): Applications, structure, and related photophysical behavior. Chem Rev. 2002;102: 759–781. doi: 10.1021/cr010142r 11890756

[10] Blattner FR, Plunkett G, Bloch CA, Perna NT, Burland V, Riley M, et al. The complete genome sequence of Escherichia coli K-12. Science (80-). 1997;277: 1453–1462. doi: 10.1126/science.277.5331.1453 9278503

[11] Zhang S, Pohnert G, Kongsaeree P, Wilson DB, Clardy J, Ganem B. Chorismate Mutase-Prephenate Dehydratase from Escherichia Coli. J Biol Chem. 1998;273: 6248–6253. doi: 10.1074/jbc.273.11.6248 9497350

[12] Ramirez MS, Tolmasky ME. Aminoglycoside modifying enzymes. Drug Resist Updat. 2010;13: 151–171. doi: 10.1016/j.drup.2010.08.003 20833577 PMC2992599

[13] Lipinszki Z, Vernyik V, Farago N, Sari T, Puskas LG, Blattner FR, et al. Enhancing the Translational Capacity of E. coli by Resolving the Codon Bias. ACS Synth Biol. 2018;7: 2656–2664. doi: 10.1021/acssynbio.8b00332 30351909

[14] Lee JK, Jeong E, Lee J, Jung M, Shin E, Kim Y hoon, et al. Directed evolution of CRISPR-Cas9 to increase its specificity. Nat Commun. 2018;9. doi: 10.1038/s41467-018-05477-x 29339724 PMC5770429

