# Supplementary material for: A bacterial dual positive and negative selection system for dCas9 activity
Source: PLoS One. 2022 Jun 3;17(6):e0269270. doi: 10.1371/journal.pone.0269270 (PMC9165777; doi:10.1371/journal.pone.0269270)
Supplement: S1 File — (PDF) [file pone.0269270.s001.pdf]

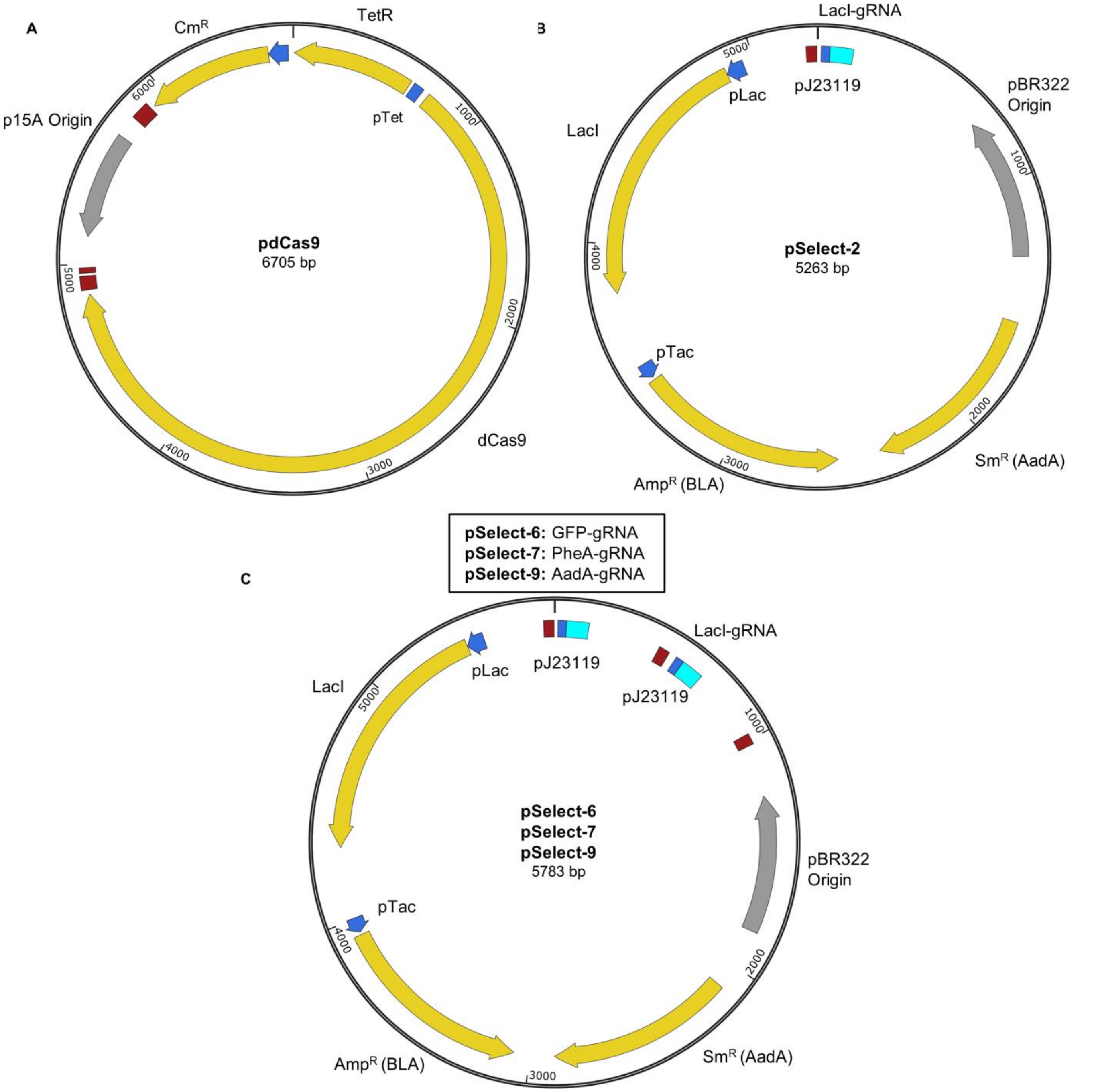

Supplementary Fig S1. Representative plasmid maps.
All proteins are shown in yellow, promoters in dark blue, origins in grey, gRNAs in teal, and terminators in red. Plasmid maps were generated using SnapGene. (A) pdCas9. (B) pSelect-2. (C) pSelect-6, pSelect-7, and pSelect-9. Each plasmid is identical except the gRNAs, as indicated.

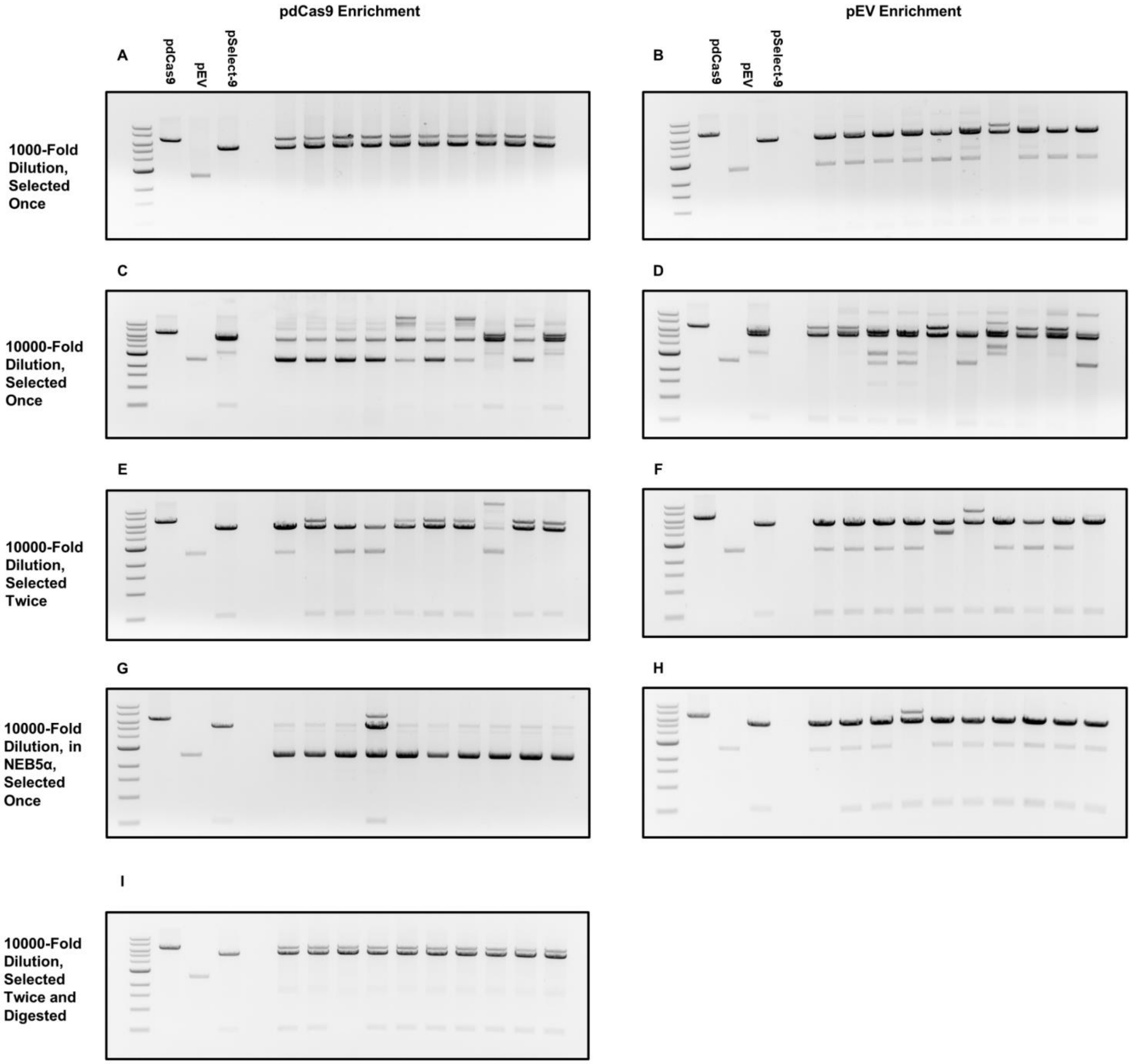

Supplementary Fig S2. Mock Enrichment of Active and Inactive dCas9.
The left column of gels (A, C, E, G, and I) contains DNA from the pdCas9 enrichment experiments (positive selections) while the right column of gels (B, D, F, and H) contains DNA from the pEV enrichment experiments (negative selections). For each gel, the first lane contained the New England Biolabs 1 kb DNA ladder. The second, third, and fourth lanes contained pdCas9, pEV, and pSelect-9 DNA, respectively. The final ten lanes contain DNA isolated from colonies picked from each experiment. The left indicates the fold-dilution of the plasmid indicated at the top and the number of times the selection was performed (serially). The strain was MG1655 except for (G) and (H). For (I), the DNA was subjected to ApaL1 and T5 exonuclease between the two selections.

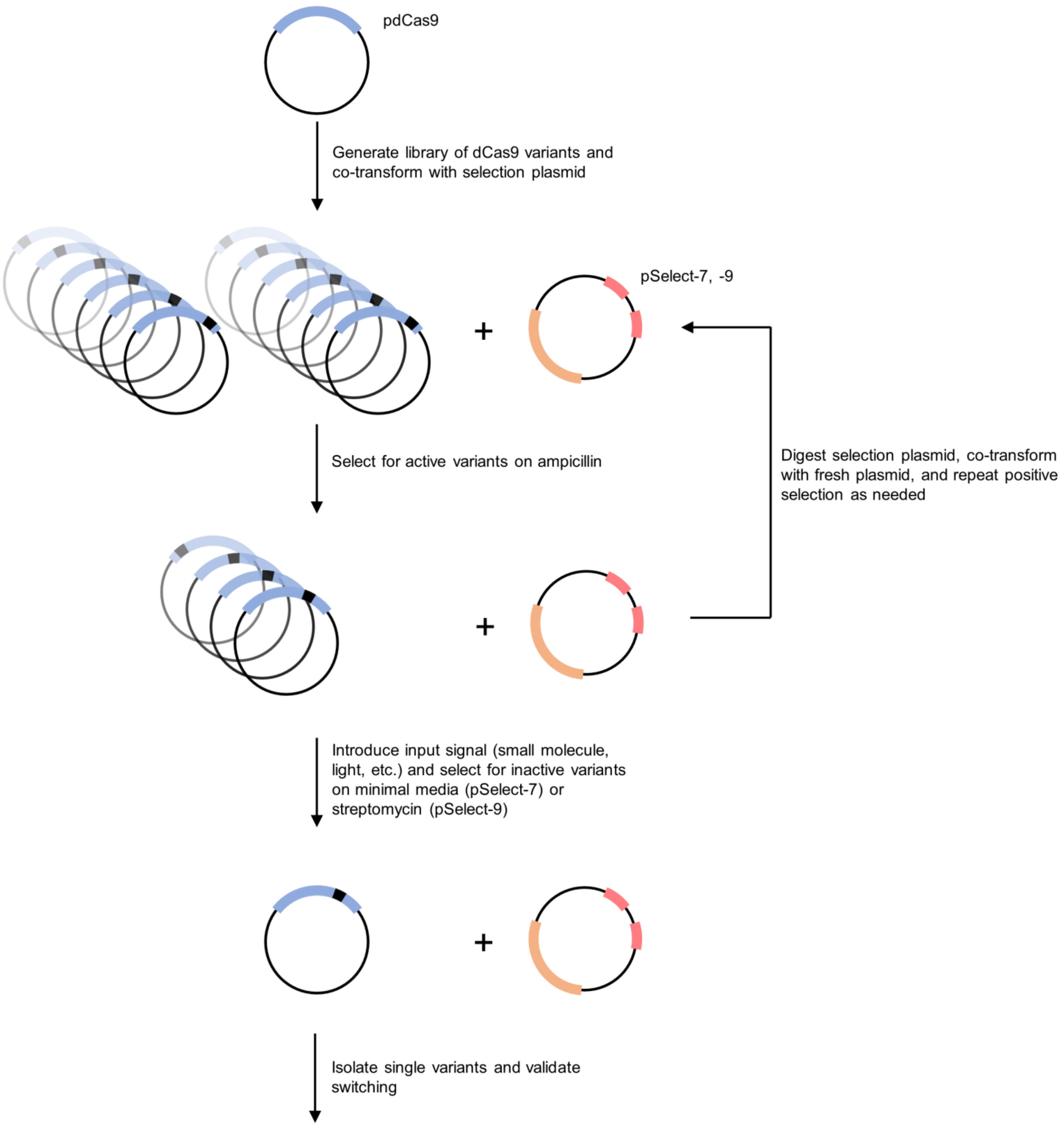

Supplementary Figure S3. Flowchart Depicting the Potential Use of the pSelect System for isolating protein switches.

Positive selection for active dCas9 variants followed by negative selection for inactive variants can be performed to identify dCas9 switches. Enrichment can be enhanced by performing multiple rounds of selections. Mutations leading to gain of Amp resistance by undesired mechanisms can be removed by an optional retransformation into fresh cells with fresh pSelect plasmid.

Supplementary Table S1. Plasmid and strain list.

**Strain**	**Description**	**Reference**
NEB5α	*fhuA2 (argF-lacZ)U169 phoA glnV44 80 (lacZ)M15 gyrA96 recA1 relA1 endA1 thi-1 hsdR17*	NEB
K12 MG1655	*F- λ- ilvG- rfb-50 rph-1*	Blattner et al. (1997) [10]
Fluorescent K12 MG1655	K12 MG1655 *nfsA::(mRFP, sfGFP,* Kan^{R}*)*	Qi et al. (2013) [3]
**Plasmids**	**Description**	**Reference**
pdCas9	Contains *TetR* and *dCas9* under control of the *Tet* promoter. *p15A* origin, Cm^{R}	Qi et al. (2013) Addgene #44249
pEV	pdCas9 backbone without the *dCas9* gene	This Study
pgRNA	Contains an *mRFP*-specific gRNA under the control of the *J23119* promoter. *pBR322* origin, Amp^{R}	Qi et al. (2013) Addgene #44251
pgRNA-LacI	pgRNA containing a *lacI*-targeting gRNA	This Study
pgRNA-GFP	pgRNA containing a *GFP*-targeting gRNA	This Study
pgRNA-PheA	pgRNA containing a *pheA*-targeting gRNA	This Study
pgRNA-SmR	pgRNA containing an *aadA*-targeting gRNA	This Study
pSKunk-BLA	Contains β-lactamase under control of the *Tac* promoter. *p15A* and *F1* origins, Sm^{R}	Firnberg et al. (2012) [13]
pgRNA-BLA	Contains origin of replication and gRNA from pgRNA-LacI and *β-lactamase* and *aminoglycoside adenlylytransferase* genes from pSkunk-BLA.	This Study
pMJ841	Contains *lacI* and a *6xHIS-MBP-dCas9* fusion under the control of the *Lac* promoter. *pBR322* and *F1* origins, Kan^{R}	Jinek et al. (2012) [2] Addgene #39318
pSelect-1	pgRNA-BLA with *lacI* from pMJ841 inserted upstream of β-lactamase.	This Study
pSelect-2	pSelect-1 with GTG to ATG mutation at the *lacI* start codon.	This Study
pSelect-6	pSelect-2 with *lacI*- and *GFP*-targeting gRNAs arranged in tandem.	This Study
pSelect-7	pSelect-2 with *lacI*- and *pheA*-targeting gRNAs arranged in tandem.	This Study
pSelect-8	pSelect-2 with an alternative *aadA*-targeting gRNA.	This Study
pSelect-9	pSelect-2 with *lacI*- and *aadA*-targeting gRNAs arranged in tandem.	This Study
