# Supplementary material for: A bacterial dual positive and negative selection system for dCas9 activity
Source: PLoS One. 2022 Jun 3;17(6):e0269270. doi: 10.1371/journal.pone.0269270 (PMC9165777; doi:10.1371/journal.pone.0269270)
Supplement: S1 Raw images — (PDF) [file pone.0269270.s003.pdf]

Fig S2A

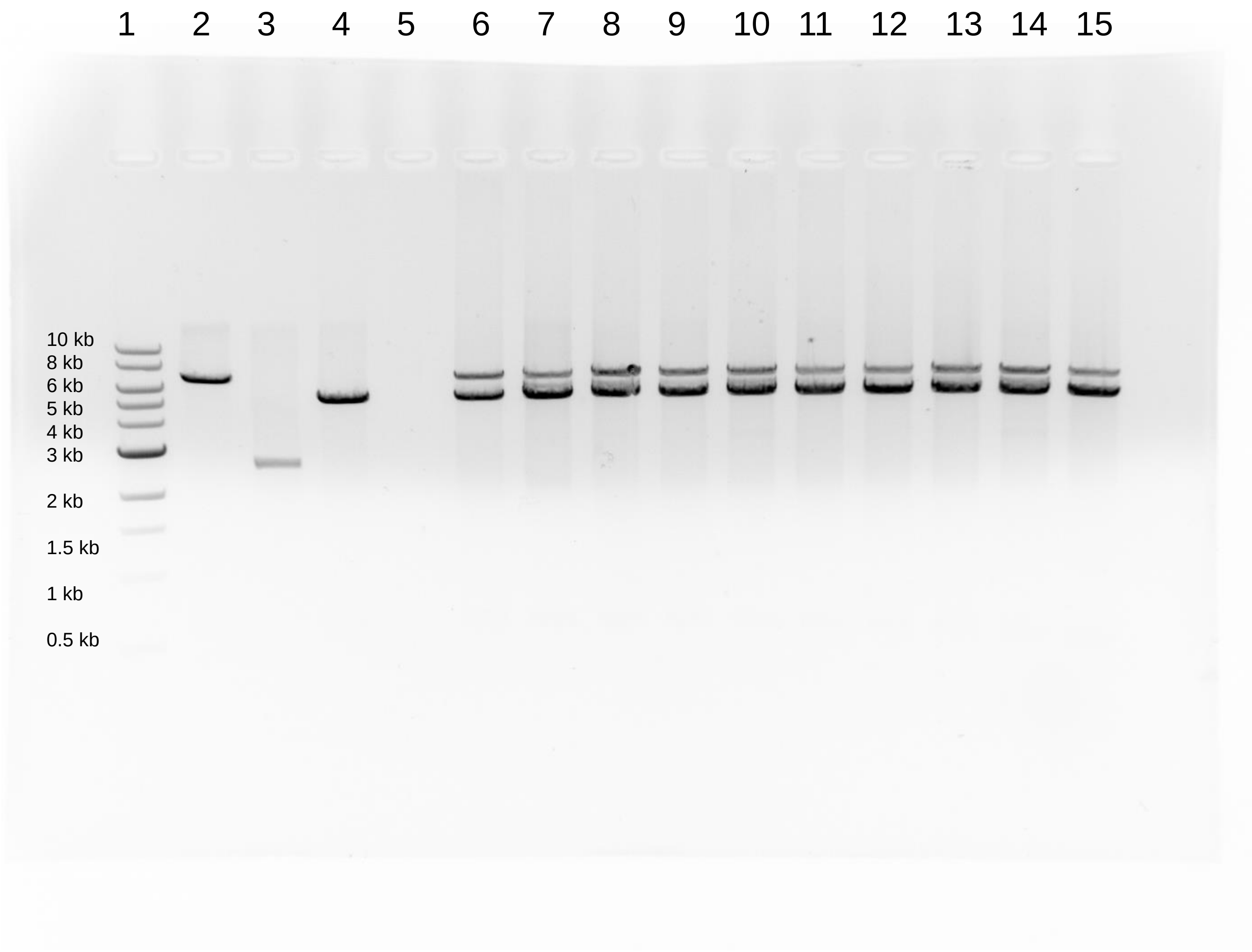

Lane Key:
1 – New England Biolabs 1 kb DNA Ladder
2 – pdCas9 plasmid DNA digested with BglII
3 – pEV plasmid DNA digested with BglII
4 – pSelect-9 plasmid DNA digested with BglII
5 – Left intentionally empty
6 through 15 – Plasmid DNA isolated from 10 bacterial colonies and digested with BglII after undergoing a single positive selection on 256 μg/mL ampicillin

Fig S2B

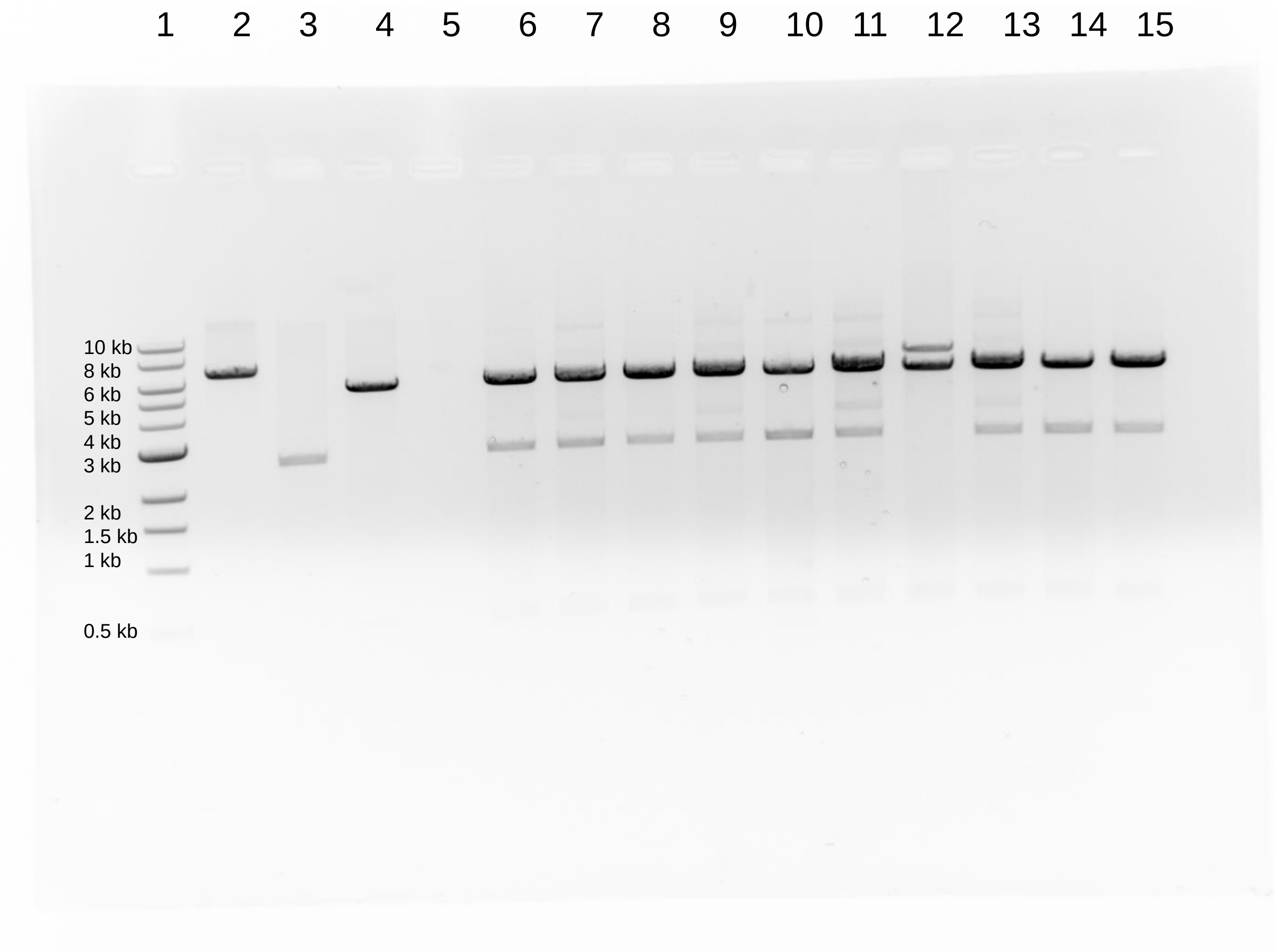

Lane Key:

1 – New England Biolabs 1 kb DNA Ladder

2 – pdCas9 plasmid DNA digested with BglII

3 – pEV plasmid DNA digested with BglII

4 – pSelect-9 plasmid DNA digested with BglII

5 – Left intentionally empty

6 through 15 – Plasmid DNA isolated from 10 bacterial colonies and digested with BglII after undergoing a single negative selection on 256 μg/mL streptomycin

Fig S2C

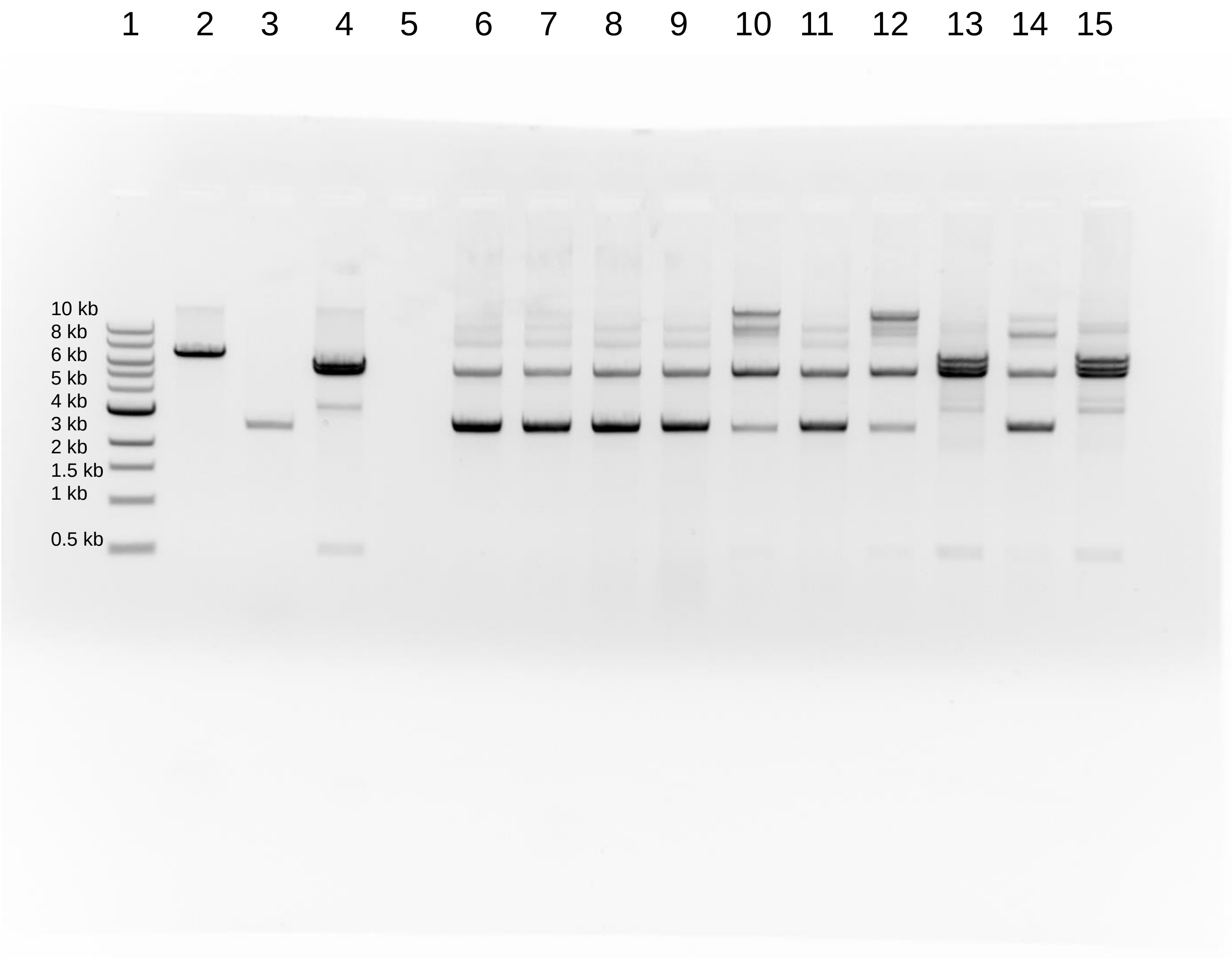

Lane Key:

1 – New England Biolabs 1 kb DNA Ladder

2 – pdCas9 plasmid DNA digested with BglII

3 – pEV plasmid DNA digested with BglII

4 – pSelect-9 plasmid DNA digested with BglII

5 – Left intentionally empty

6 through 15 – Plasmid DNA isolated from 10 bacterial colonies and digested with BglII after undergoing a single positive selection on 256 µg/mL ampicillin

Fig S2D

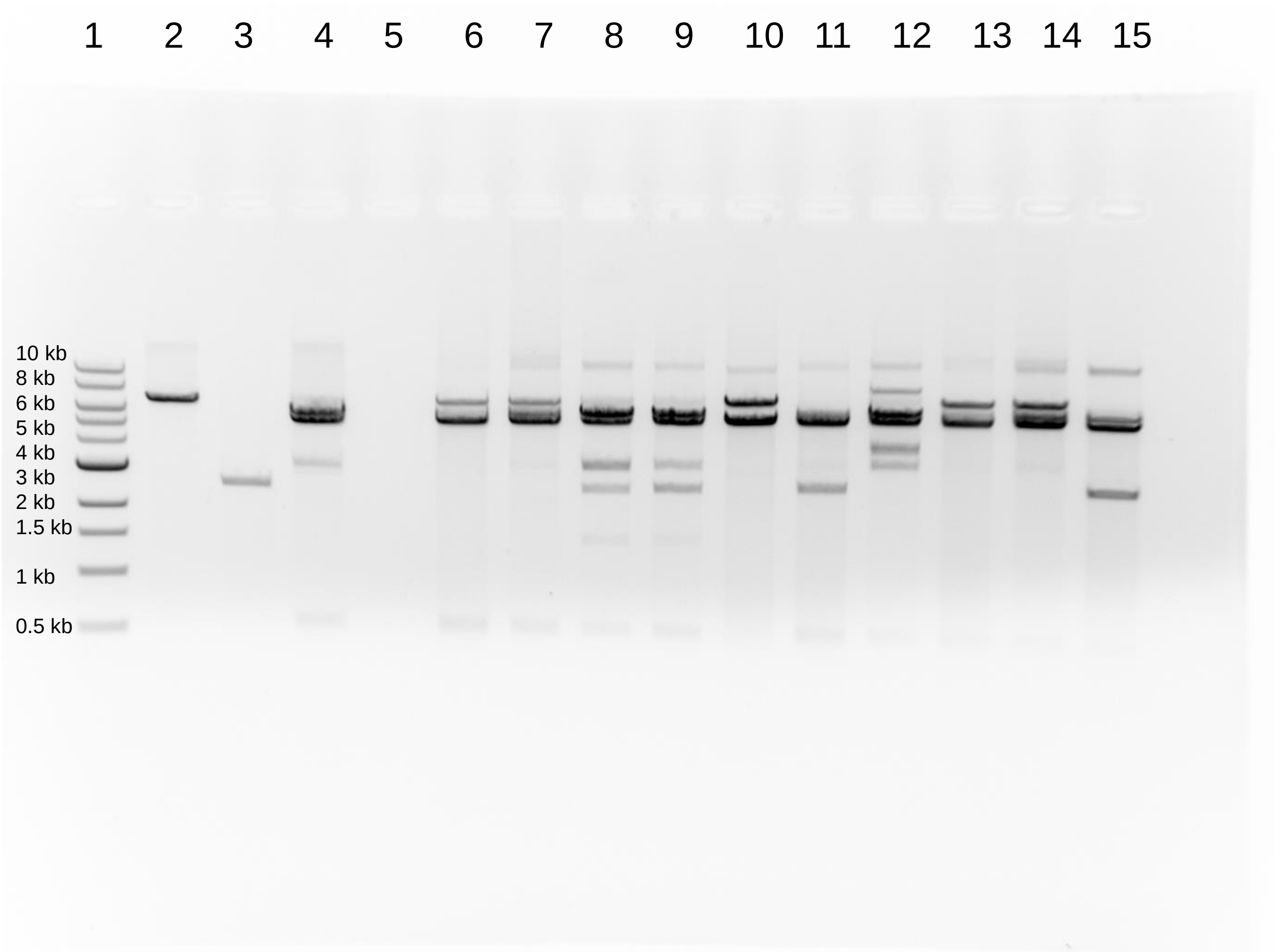

Lane Key:

1 – New England Biolabs 1 kb DNA Ladder

2 – pdCas9 plasmid DNA digested with BglII

3 – pEV plasmid DNA digested with BglII

4 – pSelect-9 plasmid DNA digested with BglII

5 – Left intentionally empty

6 through 15 – Plasmid DNA isolated from 10 bacterial colonies and digested with BglII after undergoing a single negative selection on 256 μg/mL streptomycin

Fig S2E

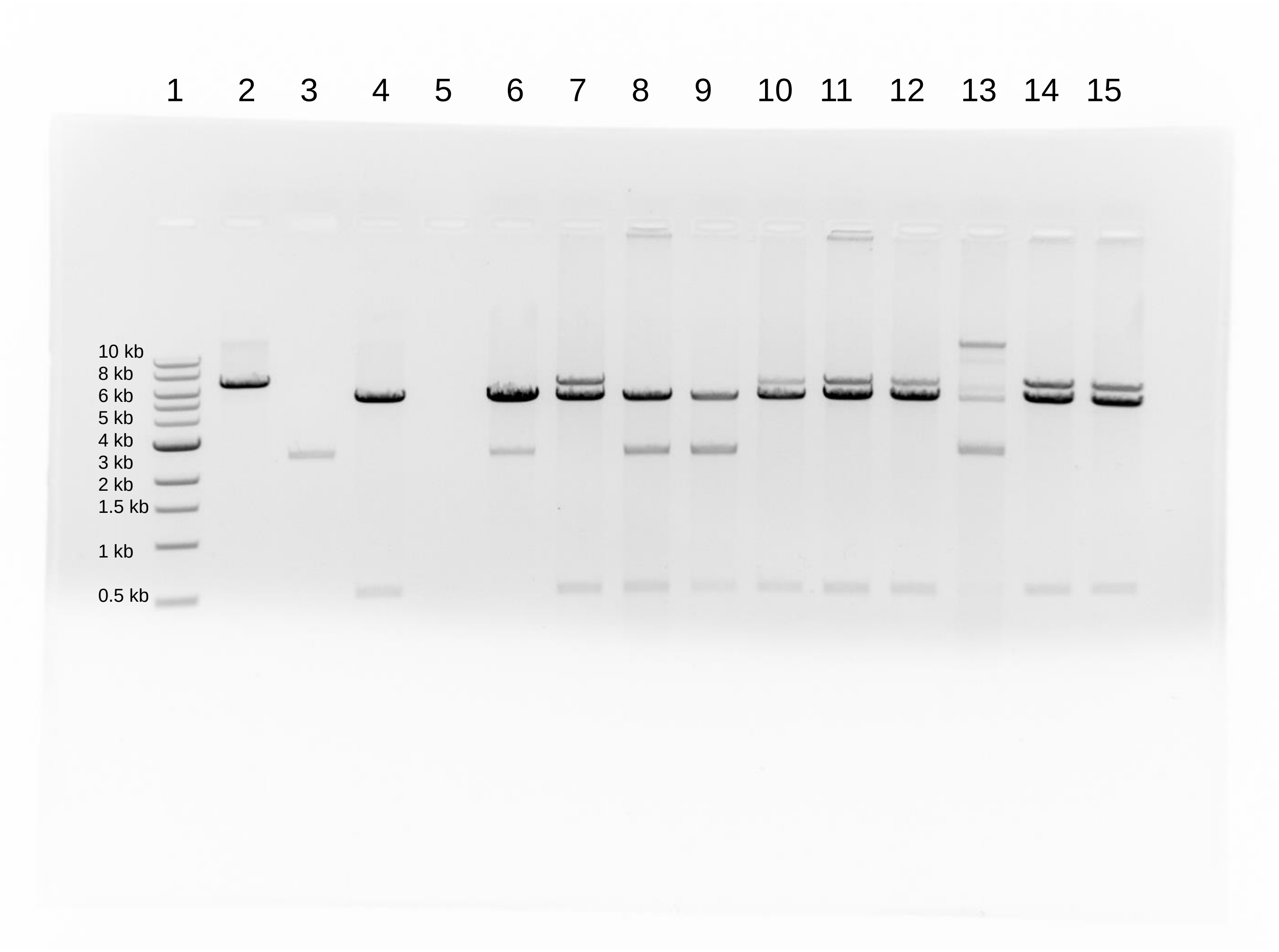

Lane Key:

1 – New England Biolabs 1 kb DNA Ladder

2 – pdCas9 plasmid DNA digested with BglII

3 – pEV plasmid DNA digested with BglII

4 – pSelect-9 plasmid DNA digested with BglII

5 – Left intentionally empty

6 through 15 – Plasmid DNA isolated from 10 bacterial colonies and digested with BglII after undergoing two positive selections on 256 µg/mL ampicillin

Fig S2F

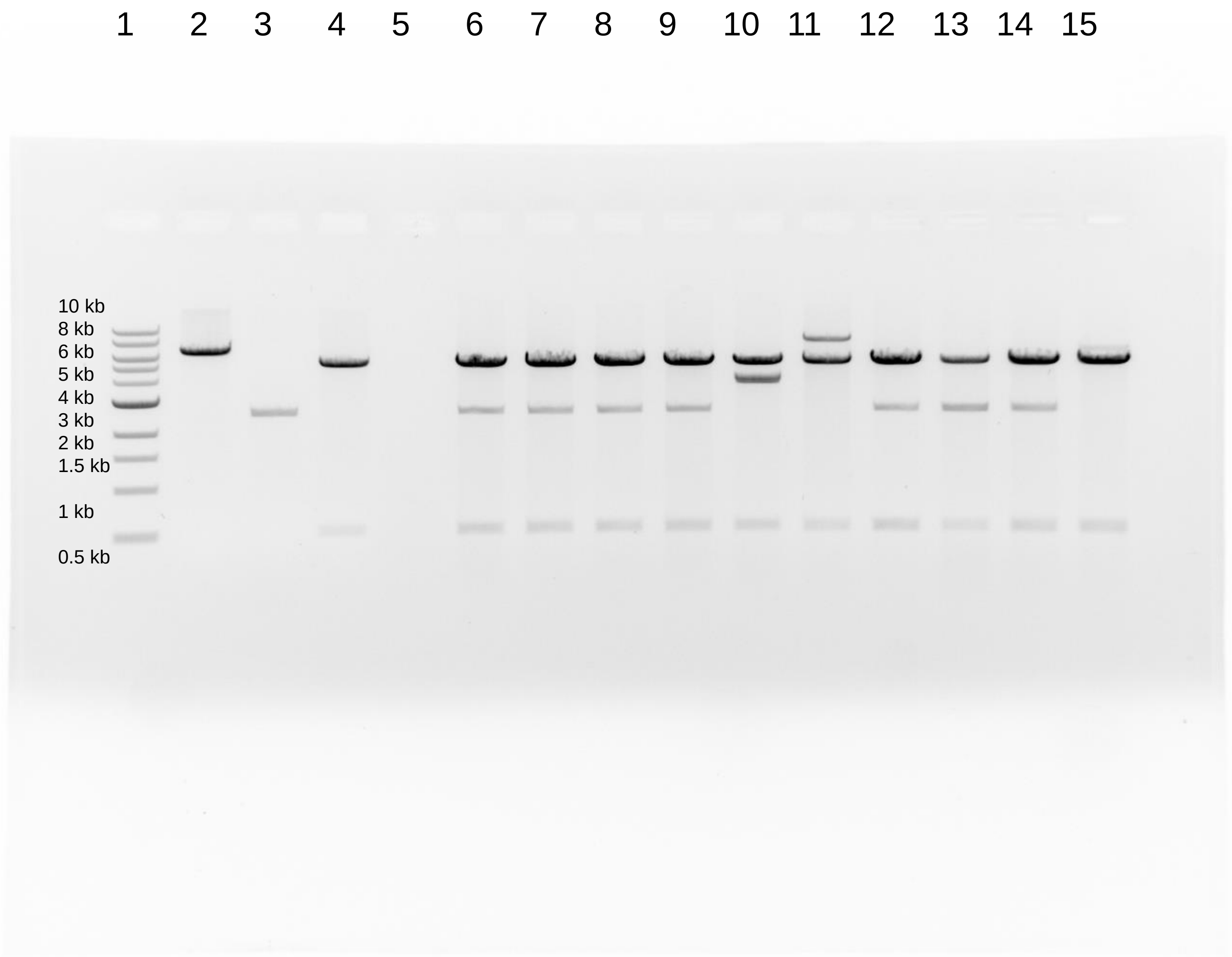

Lane Key:
1 – New England Biolabs 1 kb DNA Ladder
2 – pdCas9 plasmid DNA digested with BglII
3 – pEV plasmid DNA digested with BglII
4 – pSelect-9 plasmid DNA digested with BglII
5 – Left intentionally empty
6 through 15 – Plasmid DNA isolated from 10 bacterial colonies and digested with BglII after undergoing two negative selections on 256 μg/mL streptomycin

Fig S2G

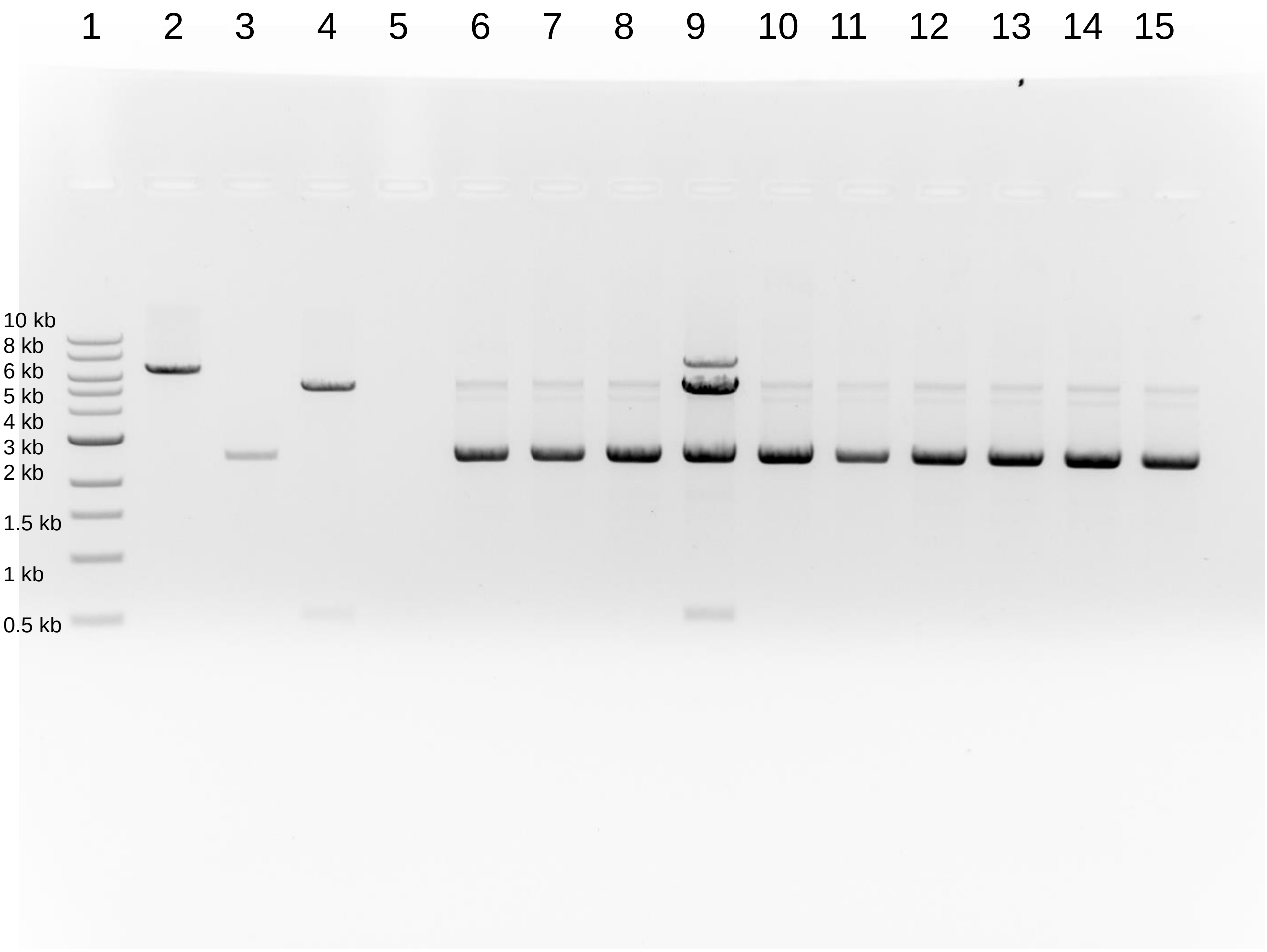

Lane Key:

1 – New England Biolabs 1 kb DNA Ladder

2 – pdCas9 plasmid DNA digested with BglII

3 – pEV plasmid DNA digested with BglII

4 – pSelect-9 plasmid DNA digested with BglII

5 – Left intentionally empty

6 through 15 – Plasmid DNA isolated from 10 bacterial colonies and digested with BglII after undergoing a single positive selection on 256 μg/mL ampicillin

Fig S2H

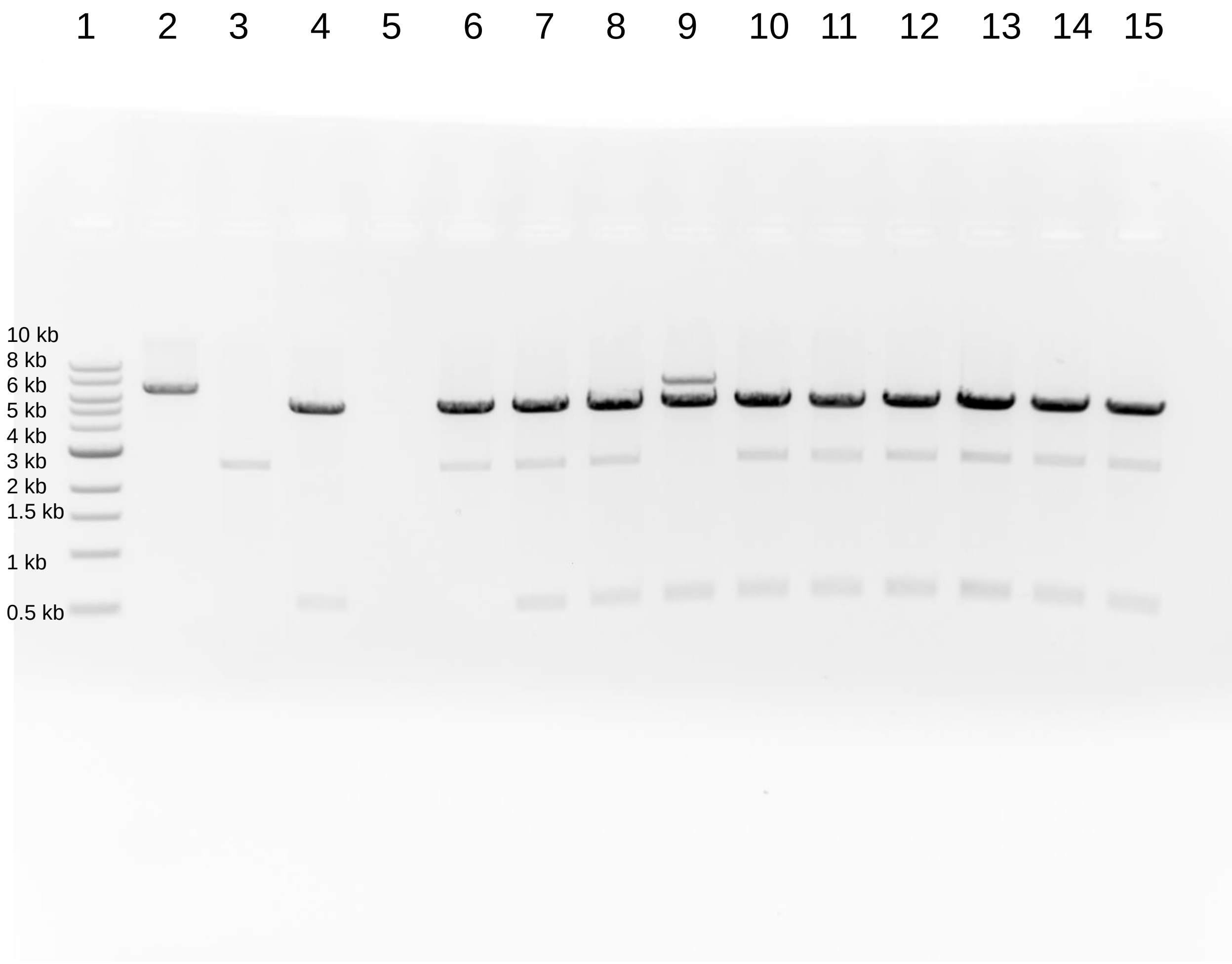

Lane Key:
1 – New England Biolabs 1 kb DNA Ladder
2 – pdCas9 plasmid DNA digested with BglII
3 – pEV plasmid DNA digested with BglII
4 – pSelect-9 plasmid DNA digested with BglII
5 – Left intentionally empty
6 through 15 – Plasmid DNA isolated from 10 bacterial colonies and digested with BglII after undergoing a single negative selection on 256 µg/mL streptomycin

Fig S2I

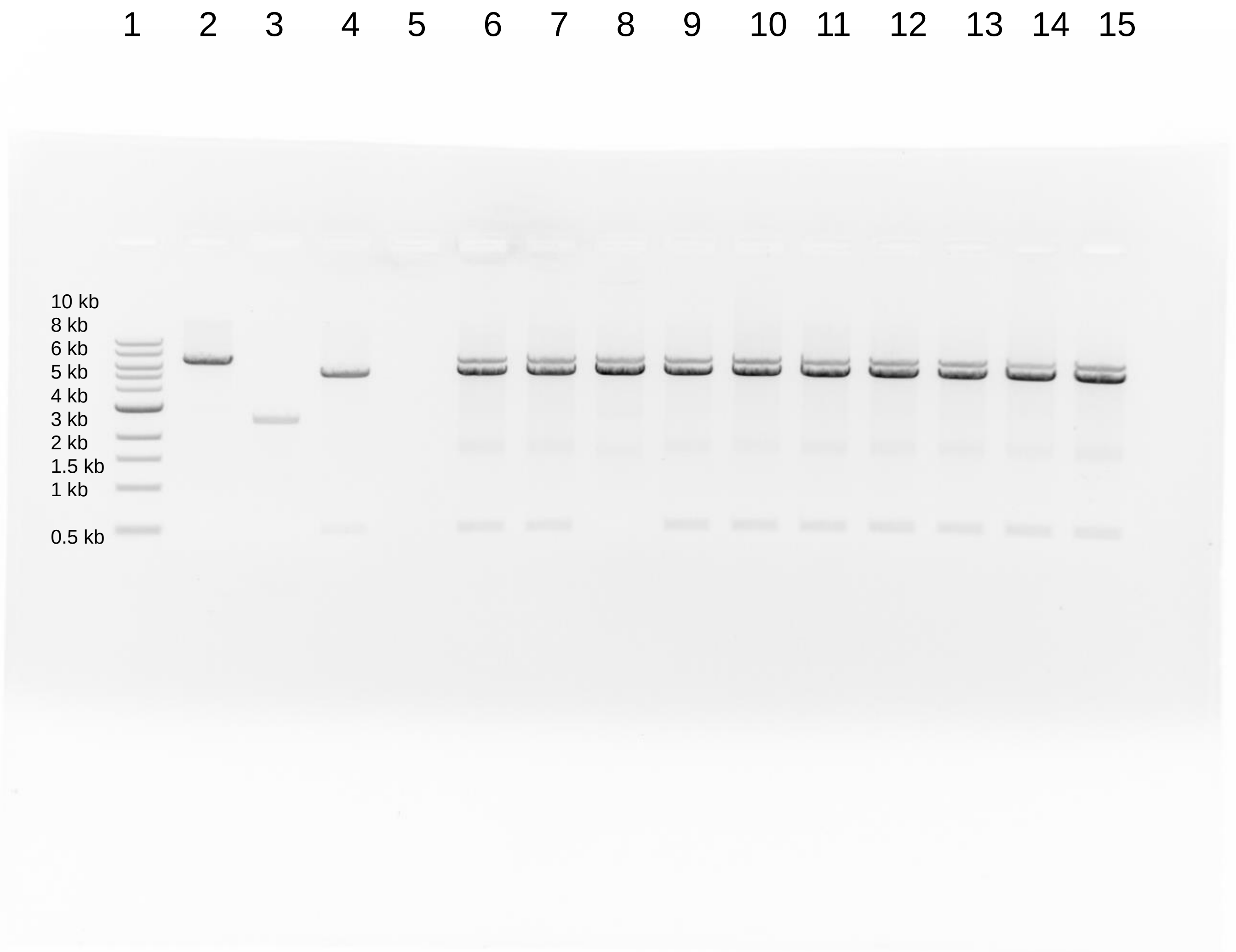

Lane Key:
1 – New England Biolabs 1 kb DNA Ladder
2 – pdCas9 plasmid DNA digested with BglII
3 – pEV plasmid DNA digested with BglII
4 – pSelect-9 plasmid DNA digested with BglII
5 – Left intentionally empty
6 through 15 – Plasmid DNA isolated from 10 bacterial colonies and digested with BglII after undergoing two positive selections on 256 µg/mL ampicillin
